# A Novel Trimethoprim Resistance Gene, *dfrA35*, Characterized from Escherichia coli from Calves

**DOI:** 10.1128/mSphere.00255-19

**Published:** 2019-05-08

**Authors:** Dominik Wüthrich, Michael Brilhante, Anna Hausherr, Jens Becker, Mireille Meylan, Vincent Perreten

**Affiliations:** a Institute of Veterinary Bacteriology, Vetsuisse Faculty, University of Bern, Bern, Switzerland; b Graduate School of Cellular and Biomedical Sciences, University of Bern, Bern, Switzerland; c Clinic for Ruminants, Vetsuisse Faculty, University of Bern, Bern, Switzerland; JMI Laboratories

**Keywords:** *Escherichia coli*, animals, antibiotic resistance, gene cassettes, trimethoprim

## Abstract

Whole-genome sequencing of trimethoprim-resistant Escherichia coli strains MF2165 and PF9285 from healthy Swiss fattening calves revealed a so far uncharacterized dihydrofolate reductase gene, *dfrA35*. Functionality and association with trimethoprim resistance were demonstrated by cloning and expressing *dfrA35* in E. coli. The DfrA35 protein showed the closest amino acid identity (49.4%) to DfrA20 from Pasteurella multocida and to the Dfr determinants DfrG (41.2%), DfrD (40.8%), and DfrK (40.0%) found in Gram-positive bacteria. The *dfrA35* gene was integrated within a florfenicol/chloramphenicol-sulfonamide resistance IS*CR2* element (*floR*-IS*CR2*-*dfrA35*-*sul2*) next to a Tn*21*-like transposon that contained genes with resistance to sulfonamides (*sul1*), streptomycin (*aadA1*), gentamicin/tobramycin/kanamycin (*aadB*), and quaternary ammonium compounds (*qacE*Δ*1*). A search of GenBank databases revealed that *dfrA35* was present in 26 other E. coli strains from different origins as well as in Acinetobacter.

**IMPORTANCE** The presence of *dfrA35* associated with IS*CR2* in Escherichia coli from animals, as well as its presence in other E. coli strains from different sources and countries and in Acinetobacter, highlights the global spread of this gene and its potential for further dissemination. The genetic link of IS*CR2*-*dfrA35* with other antibiotic and disinfectant resistance genes showed that multidrug-resistant E. coli may be selected and maintained by the use of either one of several antimicrobials.

## Abstract (precis)

The presence of *dfrA35* associated with IS*CR2* in Escherichia coli from animals, as well as its presence in other E. coli strains from different sources and countries and in Acinetobacter, highlights the global spread of this gene and its potential for further dissemination. The genetic link of IS*CR2*-*dfrA35* with other antibiotic and disinfectant resistance genes showed that multidrug-resistant E. coli may be selected and maintained by the use of either one of several antimicrobials.

## OBSERVATION

Trimethoprim is a synthetic folic acid antagonist that was introduced as an antimicrobial drug in human and veterinary medicine in the late 1960s. It is most commonly used in combination with sulfonamides: both antibiotics sequentially inhibit bacterial synthesis of tetrahydrofolic acid, which is a cofactor essential for the synthesis of thymidine and purine, the fundamental bases of DNA and RNA (1–4). Sulfonamides are analogues of *para*-aminobenzoic acid (PABA) and compete with PABA to bind to the dihydropteroate synthase (DHPS), thereby inhibiting the synthesis of dihydrofolic acid. Trimethoprim binds to the dihydrofolate reductase (DHFR), thereby blocking the conversion of dihydrofolic acid into tetrahydrofolic acid (1, 2). The use of trimethoprim/sulfonamides in both veterinary and human medicine has selected for a resistant bacterial population. In Switzerland, 28% of the Escherichia coli strains isolated from humans and up to 25% of E. coli strains from cattle and pigs have been reported to be resistant to the combination of these antimicrobials (5).

Resistance to sulfonamides and trimethoprim has been associated with five main mechanisms, including (i) a permeability barrier, (ii) a naturally insensitive intrinsic DHFR, (iii) spontaneous chromosomal mutations in the intrinsic DHPS (*folP*) and DHFR (*folA*) genes involved in the folic acid pathways, (iv) increased production of the sensitive target enzyme by upregulation of gene expression or gene duplication, and (v) the acquisition of alternative DHPS (*sul*) and DHFR (*dfr*) genes with integrons, plasmids, and transposons (6, 7). To date, three different alternative *sul* genes (*sul1*, *sul2*, and *sul3*) and 40 different types of alternative *dfr* genes have been described in Gram-positive and Gram-negative bacteria (8–10). However, the trimethoprim resistance mechanism remained unknown for 8 of 56 trimethoprim-resistant E. coli strains isolated from rectal swabs of healthy veal calves in 2017 in Switzerland (11). We selected two genetically diverse E. coli strains (MF2156 and PF9285) from two different farms based on *rep*-PCR profile to further investigate the nature of the trimethoprim resistance in these strains. The whole-genome sequences of both strains were screened for DHFR homologs followed by proof of functionality.

### Identification and localization of a new DHFR.

**FIG 1:**
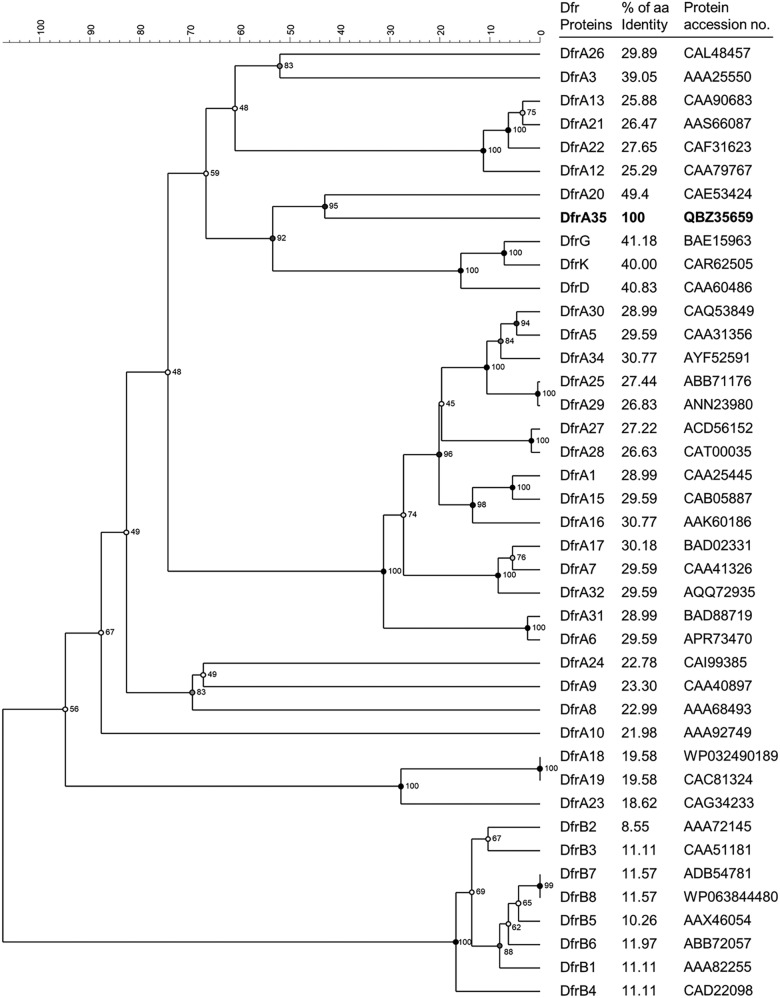
Phylogenetic tree of all known Dfr proteins, including the novel protein DfrA35. The tree was obtained by multiple alignment of amino acid sequences (without fast alignment) and the UPGMA clustering method with Jukes and Cantor correction using Bionumerics 7.6 (Applied Maths, Kortrijk, Belgium): multiple alignment with an open gap penalty (OG) of 100%, a unit gap penalty (UG) of 0%, a gap penalty of 100%, and 1,000 bootstrap values (nodes). Scala results are shown as distances. The percentages of amino acid sequence identity between DfrA35 and other Dfr proteins were determined by multiple sequence alignment with clustalW 2.1 (cost matrix Blosum) using Geneious prime 2019.1.1 (Biomatters, Ltd., Auckland, New Zealand).

Genomic DNA was extracted using the DNeasy blood and tissue kit (Qiagen, Inc., Venlo, The Netherlands), and purified using the AMPure XP PCR purification system (Beckman Coulter Life Sciences, Indianapolis, IN). DNA was sequenced on an Illumina HiSeq platform (2 × 150 paired ends) (Eurofins, Constance, Germany) and on a R9.4 SpotON flow cell and library kit (Oxford Nanopore Technologies, Oxford, United Kingdom) to obtain long reads and facilitate genome assembly. Genome assembly and read mapping of the Illumina reads against the MinION scaffolds were performed as previously described (12). Analyses of the complete genome using RESFinder 3.1 (Center for Genomic Epidemiology, DTU, Denmark) (20% coverage, 30% identity) confirmed the absence of any known acquired *dfr* gene in both strains MF2156 and PF9285. Comparison of the chromosomal *folA* gene with that of the trimethoprim-susceptible E. coli strain K-12 MG1655 (GenBank accession no. NC_000913) showed no mutation in this gene in both strains, suggesting the presence of an alternative mechanism. Search for a possible new acquired *dfr* gene within the complete genomes of MF2156 and PF9285 using blastx (https://blast.ncbi.nlm.nih.gov/) and DfrA1 (NCBI accession no. CAA25445) as the reference revealed the presence of a 177-amino-acid DHFR homologue (513 bp) in both strains. This putative new DHFR shared the closest amino acid identity (49.4%) with DfrA20 from Pasteurella multocida (GenBank accession no. CAE53424) (13) and was next closely related to Dfr proteins DfrD, DfrG, and DfrK, which have been identified so far only in Gram-positive bacteria (Fig. 1). The new gene was named *dfrA35* following the nomenclature used for Gram-negative bacteria (9).

**FIG 2:**
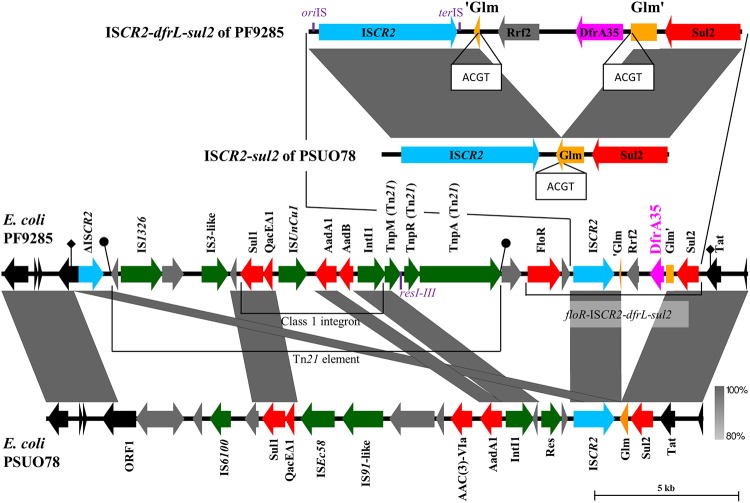
Schematic representation of the integration of *dfrA35* into IS*CR2*-*sul2* (IS*CR2*-*dfrA35*-*sul2*) and its genetic linkage with a Tn*21*-like transposon in E. coli PF9285. Shown are open reading frames (ORFs) and functions. The ORF of DfrA35 is indicated by a magenta arrow and represents the dehydrofolate reductase for trimethoprim resistance. ORFs of other antibiotic resistance proteins are in red: Sul1 and Sul2, dihydropteroate synthase for sulfonamide resistance; AadA1, streptomycin/spectinomycin 3″-adenylyltransferase ANT(3″)-Ia; AadB, aminoglycoside-2″-*O*-nucleotidyltransferase ANT(2″)-Ia; FloR, florfenicol/chloramphenicol export protein; QacEΔ1, quaternary ammonium compound efflux transporter; aminoglycoside 3-*N*-acetyltransferase. ORFs of hypothetical proteins are represented by gray arrows. ORFs of transposases associated with insertion sequences (IS), transposon Tn*21* (Tnp), as well as integrase (IntI1) and resolvase (Res) are indicated by green arrows, except for the ORFs of IS*CR2* and ΔIS*CR2*, which are indicated in deep sky blue. The *res* sites I, II, and III of Tn*21* as well as the *ori*IS and *ter*IS sequences of IS*CR2* are indicated in purple. The putative phosphoglucosamine mutase Glm as well as the 3′-end truncated part (Glm′) and the 5′-end truncated part (′Glm) are indicated in orange. ORFs representing the core genomes of strains PF9285 (GenBank accession no. CP038791) and PSUO78 (GenBank accession no. CP012112) are indicated by black arrows with Tat as a putative Tat pathway signal sequence protein and ORF1 as a putative restriction endonuclease subunit S protein. Arrows with black diamonds attached indicate the limit of the 22,977-bp region in PF9285 as determined by the end of common sequences between the E. coli PF9285 and PSUO78 chromosomes interrupted by ΔIS*CR2* on the left side and by the beginning of the *sul2* core sequences on the right side (positions 989354 to 1012331 in CP038791). Inverted repeats of the Tn*21* element are indicated by oval arrows with black circles (IV-L, GGGGGCACCTCAGAAAACGGAAAATAAAGCACGCTAAG; and IV-R, CTTAGCGTGCTTTATTTTCCGTTTTCTGAGACGACCCC). Direct repeats of ACGT duplicated by the insertion of the *rrf2*-*dfrA35* fragment into *pgm* are indicated in white boxes. The figure was created using Microsoft PowerPoint and Easyfig 2.2.2 (22).

The *dfrA35* gene was located on a 22,977-bp fragment integrated in the same location of the chromosome in both E. coli strains MF2156 and PF9285. Both ends of the integrated element were identified by comparative analysis of sequences of strains MF2156 and PF9285 with those from E. coli strain PSU078, which shared an identical flanking region (GenBank accession no. CP012112). The fragment was delimited by a truncated IS*CR2* (ΔIS*CR2*) interrupting a putative restriction endonuclease subunit S gene on one side and by a *sul2* gene interrupting a putative Tat pathway signal sequence protein gene on the other side. The fragment contains an IS*CR2* element carrying the *dfrA35* gene, the florfenicol/chloramphenicol export gene *floR*, and the sulfonamide resistance gene *sul2* (*floR*-IS*CR2-dfrA35-sul2*) and a 14,231-bp Tn*21*-like element (Fig. 2). Given the comparison to PSUO78, it is likely that the *dfrA35* region and the Tn*21*-like element were mobilized into the chromosome either simultaneously or separately by homologous recombination. The *dfrA35* gene was integrated with a gene of the Rrf2 transcriptional regulator family into the phosphoglucosamine mutase gene *glm* of a *floR*-IS*CR2-sul2* element, which has been previously reported in Stenotrophomonas maltophilia (14, 15). Integration of the *dfrA35*-*rrf2* fragment split the *glm* gene into two pieces, generating a duplication of the ACGT integration sequence (Fig. 2). The *dfrA35-rrf2* has likely been trapped by the gene-capturing machinery described for IS*CR* elements and mobilized by rolling circle transposition, but *dfrA35* does not seem to be a single cassette as many other *dfr* genes are (14, 16, 17). The Tn*21*-like element contained the characteristic features defining the Tn*21* subgroup with a transposase gene, *tnpA*, a resolvase gene, *tnpR*, in the same orientation, the *res* sites preceding *tnpR*, and two 38-bp inverted repeats at both ends (18, 19). It also contained a class I integron In290 (INTEGRALL database), including the integrase gene *intI1*, the sulfonamide resistance gene *sul1*, the streptomycin/spectinomycin resistance gene *aadA1* [*ant(3′')-Ia*], the gentamicin/tobramycin/kanamycin resistance gene *aadB* [*ant(2'')-Ia*], the quaternary ammonium compound (QAC) efflux transporter gene *qacE*Δ*1*, as well as the transposase genes of IS*1326* and of a new transposase gene related to IS*3*. Duplication of IS*CR2* sequences suggests that insertion and movement of the different pieces of the element may have also occurred by homologous recombination (Fig. 2).

As expected by its chromosomal location and genetic context, transferability of the *dfrA35* gene could not be observed experimentally by filter mating using strains MF1256 and PF9285 as donors and the rifampin-, sodium azide-resistant strain E. coli J53dR as the recipient (20). Selection was performed on Mueller-Hinton (MH) agar plates containing trimethoprim (30 µg/ml) with either sodium azide (100 µg/ml) or rifampin (50 µg/ml) for 48 h at 37°C.

### Expression of *dfrA35* in E. coli.

Functionality and association of *dfrA35* with trimethoprim resistance were determined by cloning an 878-bp region containing the 513-bp *dfrA35* gene and a 265-bp region upstream of the *dfrA35* start codon. This fragment was amplified by PCR using Phusion high-fidelity DNA polymerase (Thermo Fisher Scientific, Waltham, MA) and primers dfrA35-F (5′-TGGTGCGCAAATATTTCGGC-3′) and dfrA35-R (5′-ACGTTAACCCGAAAAAGCGA-3′) (annealing temperature, 56°C; extension time, 30 s) and cloned into the PCR-Blunt II-TOPO vector following the manufacturer’s instructions (Zero blunt TOPO PCR cloning kit; Thermo Fisher Scientific).

The ligated vector insert DNA was transformed into chemically competent cells of E. coli One Shot TOP10 (Thermo Fisher Scientific). Transformants obtained on LB plates containing 50 µg/ml kanamycin after 24 h of incubation at 37°C were tested for the presence of the *dfrA35* gene by PCR using *Taq* polymerase and internal primers dfrA35int-F (5′-GCATTTACCGGCCGATATGC-3′) and dfrA35int-R (5′-ACACGCAGCACCTCTTCATT-3′) (annealing temperature, 56°C; extension time, 30 s). The resulting *dfrA35*-containing plasmids, pT2156c19 and pT9285c17, were isolated using the PureLink Quick plasmid miniprep kit (Thermo Fisher Scientific) and analyzed by Sanger sequencing to confirm the veracity of the sequence and that *dfrA35* was inserted in the opposite direction of the TOPO promoter P_lac_ and was therefore under the control of its own promoter. The MIC of trimethoprim for the parent strains MF2156 and PF9285, recipient strain TOP10, and transformant strains TOP10/pT2156c19 and TOP10/pT9285c17 was determined on a microtiter plate using the 2-fold-dilution technique (concentration range, 1 to 512 µg/ml) following CLSI guidelines (21). The MIC for trimethoprim increased in E. coli TOP10 expressing *dfrA35* to 128 µg/ml compared to the nontransformed TOP10 strain (≤0.25 µg/ml), almost reaching the level of the MIC observed in the parent strains MF2156 and PF9285 (256 µg/ml). The MIC of 13 other antibiotics was determined using EUVSEC Sensititre plates (Thermo Fisher Scientific), showing an association between the other antibiotic resistance genes found on strains MF2156 and PF9285 and their phenotype (Table 1).

**TABLE 1:** Characteristics of Escherichia coli strains and MICs of antibiotics

E. coli strain (sequence type)	Origin and characteristics	Reference	Antibiotic resistance gene(s)^*a*^	MIC (µg/ml) of^*b*^:													
				AMP	AZI	CHL	CIP	CST	FOT	GEN	MERO	NAL	SMX	TAZ	TET	TGC	TMP
MF2156 (ST3057)	Rectal swab of calf 1, farm 1	This study	*aadA1*, *aadB*, *bla*_TEM-1D_, *dfrA35*, *floR*, *sul1*, *sul2*, *tet*(A), *qacE*Δ*1*	>64	≤2	128	≤0.015	≤1	≤0.25	8	≤0.03	≤4	>1,024	≤0.5	>64	0.5	**256**
PF9285 (ST10)	Rectal swab of calf 1, farm 2	This study	*aadA1*, *aadB*, *aph*(*3*′)-*Ia*, *bla*_TEM-1B_, *catA1*, *dfrA35*, *floR*, *strA*, *strB*, *sul1*, *sul2*, *tet*(A), *qacE*Δ*1*	>64	4	>128	≤0.015	≤1	≤0.25	8	≤0.03	≤4	>1,024	≤0.5	32	≤0.25	**256**
TOP10	OneShot TOP10 cells for transformation	Thermo Fisher Scientific	None	2	4	≤8	≤0.015	≤1	≤0.25	≤0.5	≤0.03	≤4	≤8	≤0.5	≤2	≤0.25	**≤0.25**
TOP10/pT2156c19	TOP10 with *dfrA35* from MF2156 cloned into vector pCR-BluntII-Topo	This study	*dfrA35*	2	4	≤8	≤0.015	≤1	≤0.25	≤0.5	≤0.03	≤4	≤8	≤0.5	≤2	≤0.25	**128**
TOP10/pT9285c17	TOP10 with *dfrA35* from PF9285 cloned into vector pCR-BluntII-Topo	This study	*dfrA35*	2	4	≤8	≤0.015	≤1	≤0.25	≤0.5	≤0.03	≤4	≤8	≤0.5	≤2	≤0.25	**128**

a Genes and functions: *aadA1*, streptomycin/spectinomycin adenyltransferase; *aadB*, gentamicin/tobramycin/kanamycin nucleotidyltransferase; *aph(3′)-Ia*, kanamycin/neomycin/paromomycin/ribostamycin/lividomycin/gentamicin B phosphotransferase; *bla*_TEM-1B_ and *bla*_TEM-1D_, ampicillin β-lactamase; *catA1*, chloramphenicol acetyltransferase; *dfrA35*, trimethoprim dihydrofolate reductase; *floR*, florfenicol/chloramphenicol exporter; *strA*, *strB*, streptomycin phosphotransferase; *sul1* and *sul2*, sulfonamide dihydropteroate synthase; *tet*(A), tetracycline exporter; *qacE*Δ*1*, quaternary ammonium compound multidrug exporter.

b Antibiotics: AMP, ampicillin; AZI, azithromycin; CHL, chloramphenicol; CIP, ciprofloxacin; CST, colistin; FOT, cefotaxime; GEN, gentamicin; MERO, meropenem; NAL, nalidixic acid; SMX, sulfamethoxazole; TAZ, ceftazidime; TET, tetracycline; TGC, tigecycline; TMP, trimethoprim. Note that the MICs for TMP have been highlighted in boldface.

### Spread of *dfrA35* in association with IS*CR2* and *sul2.*

PCR screening of 8 additional strains from calves with no known trimethoprim resistance gene revealed *dfrA35* in 5 of them. The gene was also located in these 5 strains on an IS*CR2-dfrA35-sul2* fragment, as determined by *Taq* PCR using primers ISCR2-F (5′-CGCCTGCATTGAAGACCCTA-3′) and Sul2-F (5′-TGTCTGTTTCGCGCAAATCC-3′) (annealing temperature, 56°C; extension time, 3 min). Searching for DfrA35 in the NCBI database using blastp revealed its presence in 26 other E. coli strains as well as in an Acinetobacter sp. strain originating from different sources (cattle, dogs, a horse, and humans) and countries, which indicates that the *dfrA35* gene has potential for dissemination among bacterial species even if transfer could not be demonstrated experimentally. In 18 of them, the available sequences allowed us to determine that *dfrA35* was also linked to *sul2*, and 6 sequences revealed the presence of the IS*CR2-dfrA35-sul2* element (see Table S1 in the supplemental material).

10.1128/mSphere.00255-19.1 TABLE S1 List of sequenced bacteria containing *dfrA35* found in the NCBI GenBank database (accessed 30 November 2018). Download Table S1, PDF file, 0.03 MB. Copyright © 2019 Wüthrich et al. 2019 Wüthrich et al. This content is distributed under the terms of the Creative Commons Attribution 4.0 International license.

This study identified a novel functional trimethoprim resistance gene (*dfrA35*) in E. coli from calves. This gene appeared to be widespread in other E. coli strains as well as in Acinetobacter, where it was also mainly directly flanked by IS*CR2* and/or *sul2*. In the two analyzed E. coli strains from cattle, the IS*CR2-dfrA35-sul2* sequence was also linked to *floR* and was part of a larger multiple antibiotic and QAC resistance element. The dissemination of this element may further jeopardize the efficacy of antibiotics and disinfectants in both veterinary and human medicine.

### Accession number(s).

The complete chromosome of E. coli strain PF9285 containing the 22,977-bp fragment carrying *dfrA35* and flanking regions (positions 989354 to 1012331) has been deposited in GenBank under accession no. CP038791 (BioProject no. PRJNA530748).
