# Correction for Wüthrich et al., “A Novel Trimethoprim Resistance Gene, *dfrA36*, Characterized from Escherichia coli from Calves”

**DOI:** 10.1128/mSphere.00390-19

**Published:** 2019-06-19

**Authors:** Dominik Wüthrich, Michael Brilhante, Anna Hausherr, Jens Becker, Mireille Meylan, Vincent Perreten

**Affiliations:** a Institute of Veterinary Bacteriology, Vetsuisse Faculty, University of Bern, Bern, Switzerland; b Graduate School of Cellular and Biomedical Sciences, University of Bern, Bern, Switzerland; c Clinic for Ruminants, Vetsuisse Faculty, University of Bern, Bern, Switzerland

## AUTHOR CORRECTION

Volume 4, no. 3, e00255-19, 2019, https://doi.org/10.1128/mSphere.00255-19. The *dfrA35* gene originally described in this publication has been reclassified as *dfrA36* (GenBank accession no. CP038791). The gene name has been corrected, including in the title and the supplemental material, throughout the current version of the paper, posted on 18 June 2019.

