# Supplementary material for: A Novel Trimethoprim Resistance Gene, dfrA35, Characterized from Escherichia coli from Calves
Source: mSphere. 2019 May 8;4(3):e00255-19. doi: 10.1128/mSphere.00255-19 (PMC6506621; doi:10.1128/mSphere.00255-19)
Supplement: TABLE S1 [file mSphere.00255-19-st001.pdf]

Supplementary Table S1. List of sequenced bacteria containing *dfrA35* found in the NCBI GenBank database (accessed 30 November 2018).

Species	Strain	Origin	Country	Length in aa	Identity in % aa	Identity in % nt	NCBI Acc. No,	size of overlapping region (identity)	IS*CR2-dfrA35-sul2*
E. coli	2013C-4491	human	unknown	170	100	100	CP027355	48.7 kb (99%) complete	IS*CR2-dfrA35-sul2*
E. coli	RiKo 2308/09 RIKO2308_155c	unknown	unknown	170	100	100	LCVI01000155	48.7 kb (99%) complete	IS*CR2-dfrA35-sul2*
E. coli	364072-17	unknown	Switzerland	170	100	100	PYRP01000006	24.3 kb (99%)	'IS*CR2-dfrA35*-NSA
E. coli	364062-17	unknown	Switzerland	170	100	100	PYSC01000001	24.3 kb (99%)	'IS*CR2-dfrA35*-NSA
E. coli	364068-17	unknown	Switzerland	170	100	100	PYRX01000002	24.2 kb (99%)	'IS*CR2-dfrA35-sul2*
E. coli	ecoli006	human	unknown	170	100	100	UWVI01000001	23.8 kb (99%)	IS*CR2-dfrA35-sul2*
E. coli	NT2	cattle	Czech Republic	170	100	100	QYZN01000009	23.8 kb (99%)	IS*CR2-dfrA35-sul2*
E. coli	22593	cattle	France	170	100	100	LMBK01000112	21 kb (99%)	'IS*CR2-dfrA35-sul2*
E. coli	MS 124-1	human	unknown	170	100	100	ADWT01000099	6 kb (100%)	NSA-*dfrA35-sul2*
E. coli	G76	mastitis/cattle	Germany	170	100	100	LOPR01000045	5.8 kb (100%)/1.2 kb (84%)	NSA-*dfrA35-sul2*
E. coli	RiKo 2305/09 RIKO2305_49c	unknown	unknown	170	100	100	JYPB01000049	5.8 kb (99%)	NSA-*dfrA35-sul2*
E. coli	GER_MD02_1511_Eco_064	fecal/dog	Germany	170	100	100	PURJ01000057	5.8 kb (100%)	NSA-*dfrA35-sul2*
E. coli	GER_MD06_1511_Eco_062	fecal/dog	Germany	170	100	100	PURL01000057	5.8 kb (100%)	NSA-*dfrA35-sul2*
E. coli	MS 84-1	human	unknown	170	100	100	ADTK01000026	5.5 kb (99%)	NSA-*dfrA35-sul2*
Acinetobacter sp	ANC 5318	nose/horse	Czech Republic	170	100	100	PGPB01000023	3 kb (100%)/1kb (100%)	'IS*CR2-dfrA35*-NSA
E. coli	MS 85-1	human	unknown	170	100	100	ADWQ01000115	2.3 kb (99%)	NSA-*dfrA35-sul2*'
E. coli	364073-17	unknown	Switzerland	170	100	100	PYRU01000163	2.3 kb (100%)	NSA-*dfrA35-sul2*'
E. coli	GER_MD08_1505_Eco_019	fecal/dog	Germany	170	100	100	PUSY01000104	2.3 kb (100%)	NSA-*dfrA35-sul2*'
E. coli	GER_MD08_1505_Eco_035	fecal/dog	Germany	170	100	100	PUSL01000111	2.2 kb (100%)	NSA-*dfrA35-sul2*'
E. coli	RiKo 2340/09 RIKO2340_126c	unknown	unknown	170	100	100	LAGW01000126	2.1 kb (100%)	NSA-*dfrA35-sul2*'
E. coli	G213	mastitis/cattle	Israel	170	100	100	LOOH01000094	2.1 kb (100%)	NSA-*dfrA35-sul2*'
E. coli	STEC 514-2 514-2	calf	Israel	170	100	100	MRVZ01000194	1.8 kb (100%)	NSA-*dfrA35*-NSA
E. coli	364064-17	unknown	Switzerland	170	100	100	PYSA01000272	1.8 kb (100%)	NSA-*dfrA35*-NSA
E. coli	STEC 573-4 573-4	cow	Israel	170	100	100	MRWA01000125	1.7 kb (100%)	NSA-*dfrA35*-NSA
E. coli	31_Esco_HA-NL 130741A	human	Netherlands	170	100	100	NXOD01000140	1.6 kb (100%)	NSA-*dfrA35*-NSA
E. coli	RefSeq.	hospital	unknown	170	100	100	WP_024198497	no DNA sequence	no DNA sequence
*Gammaproteobacteri*a	RefSeq.	unknown	unknown	170	100	100	WP_000949574	no DNA sequence	no DNA sequence

' : truncated NSA: No sequence available due to termination of the contig
